# Improvement in a human IgE-inducing system by *in vitro *immunization

**DOI:** 10.1186/1753-6561-7-S6-O4

**Published:** 2013-12-04

**Authors:** Shuichi Hashizume, Hiroharu Kawahara

**Affiliations:** 1 Idea-Creating Lab, Yokohama 236-0005, Japan; 2 Kitakyushu National College of Technology, Kitakyushu 802-0985, Japan

## Introduction

The immune system, which is the self-defense system of the body, occasionally responds in a manner that is harmful to the body. The incidence and severity of allergies caused by cedar pollen, house dust, egg protein, and many others are increasing and have recently become a serious social problem. We have previously developed an original *in vitro *system for inducing human IgE antibody specific to a designated antigen that can be used to study various allergic reaction [1]. In this study, we attempted to improve this system to stimulate IgE levels in its medium to provide a highly sensitive screening method.

## Experimental

The original *in vitro *IgE-inducing system was established using lymphocytes and plasma from donors which were not naturally immunized with allergens. The original system contained ERDF supplemented with fetal bovine serum (final concentration, 5%) and contained human plasma (10%) as an essential component. Human peripheral blood lymphocytes and plasma were obtained by density-gradient centrifugation at 400 × g for 30 min with cell separation medium, Ficoll-Paque™ Plus. This system also included allergen (100 ng/ml), interleukins (IL-) 2, 4, and 6 (10 ng/ml each) and muramyl dipeptide (MDP, 10 μg/ml), as described previously [2]. Human lymphocytes were cultured in 96- or 24-well plates at a final density of 1 × 10^6 ^cells/ml in the medium and incubated in a CO_2 _incubator at 37°C for 10 days. During the 10 days, IgE was specifically secreted into the medium.

## Results and discussion

### Effects of human plasma and interleukins on human IgE induction

The necessity for inclusions of human plasma and interleukins was shown, when human lymphocytes and plasma from donors which were not naturally immunized with allergens were used. For the induction of IgE, human lymphocytes and plasma obtained from the same donor were required [2]. Addition of IL-2, 4 and 6 induced IgE. Elimination of each of these three interleukins from the medium resulted in no induction of IgE (data not shown). From these results, IL-2, 4 and 6 are considered to be essential factors to initially immunize lymphocytes with allergens, when lymphocytes and plasma from donors not naturally immunized with allergens were used. We next attempted to improve this system to stimulate IgE levels in the medium to provide a highly sensitive screening method.

### Effects of elimination of IL-2 from the medium on human IgE production

In this study, the lymphocytes and plasma of donors naturally immunized with various allergens were used. Therefore, the IgE level of the control was high, i.e., more than 300 ng/ml, as shown in Table 1. Elimination of IL-2 from the medium resulted in the induction of higher IgE levels compared with medium containing IL-2 (Table 1). These data indicate that elimination of IL-2 from the medium induced higher IgE levels when human lymphocytes and plasma obtained from naturally immunized donors were used. Furthermore, strawberry extract in the media containing Cryj1 and Derf2 decreased the secreted IgE levels by 38% and 24%, respectively. There is a possibility that strawberries may alleviate allergies.

**Table 1:** Effects of various additives on IgE productivity

Medium	IgE productivity (ng/ml)
Control (ERDF + hPlasma + FBS)	319 ± 19

+ IL-2 + IL-4 + IL-6 + MDP + Cryj1	356 ± 85

+ IL-4 + IL-6 + MDP + Cryj1	549 ± 189

+ IL-4 + IL-6 + MDP + Cryj1 + strawberry extract	341 ± 55

+ IL-4 + IL-6 + MDP + Derf2	660 ± 172

+ IL-4 + IL-6 + MDP + Derf2 + strawberry extract	499 ± 167

In summary, elimination of IL-2 from the IgE-inducing system medium increased the IgE induction level when human lymphocytes and plasma obtained from donors naturally immunized with allergens were used. The level of about 1 μg/ml IgE reported to be secreted in this study may be the highest compared with those reported elsewhere. The original and improved systems for human IgE production are considered to be of profound use for studying allergy mechanisms and surveying allergy-alleviating products, respectively.
